# Model of organizational stress for use within an occupational health education/promotion or well-being of members of the organization

**DOI:** 10.4103/0972-6748.62276

**Published:** 2009

**Authors:** Subhash S. Sharma

**Affiliations:** Department of Psychology, Bhavnagar University, Bhavnagar, India

**Keywords:** Health and safety executive, Risk assessment, Stress management, Stress prevention, Work-related stress

## Abstract

This paper introduces a simple model of organizational stress which can be used to educate or inform employees, personnel and health professionals about the relationship between potential work-related stress hazards, individual and organizational symptoms of stress, negative outcomes and financial costs. The components of the model relate directly to a recent Health and Safety Executive publication (Cox, 1993) which focuses on improving and maintaining employee health and well-being.

During the 1990s, the Health and Safety Executive (HSE) published a number of documents which provided information for health and safety practitioners and employers on stress, stress research and stress prevention (Cox, 1993; Cox, 1995; Cox, 1998; Cox, 2000). However, the guidance for employers lacked a structured approach for work-related stress prevention programs although the publications highlighted many possible strategies, such as staff training and improving communication (Pestonjee, 1999; Ram, 1998; Joshi, 2007). In 2001, the HSE published a guide for managers running units or departments with 50 or more staff (Cox, 2001). The guide focused on the application of a structured approach to stress prevention by providing a 5-step work-related stress risk assessment to aid diagnosis of the problem(s) and provide a framework for intervention. Unfortunately, from an educational viewpoint, the document did not include a model of stress to underpin the theory and practice advocated by the document. To help explain the new guidance to managers, personnel and health professionals, the authors of this paper developed a simple model of stress, based on earlier ‘engineering’ models of stress (Sutherland and Cooper, 2001), which includes the main stress-related hazards and outcomes discussed in the HSE document. This model of stress was later launched at a major annual conference of personnel and development professionals.

## MODEL OF STRESS

**Figure 1:**
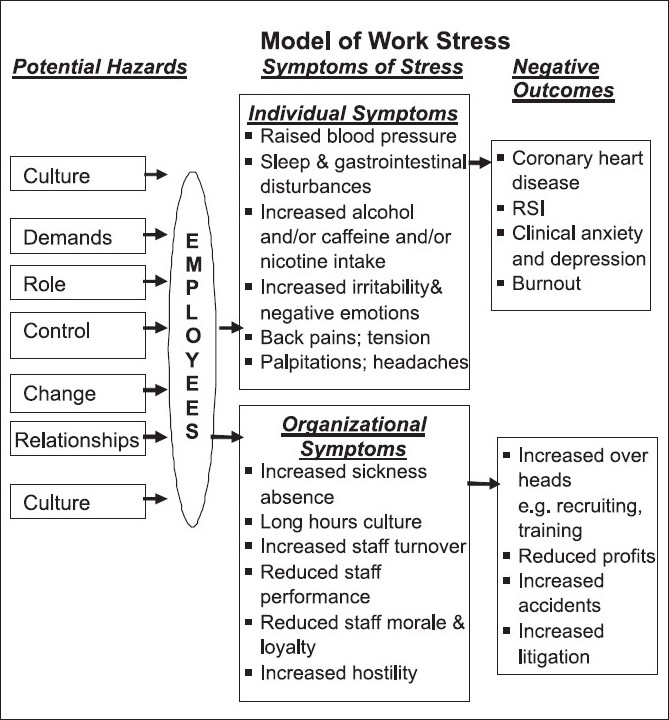
Model of work stress

The overall model of stress [Figure 1] is self-explanatory. However, in the authors’ experience, the HSE have chosen 7 key hazards that usually require further explanation depending upon the level of knowledge of the employees or professionals involved (see below). It is worth noting that 1995-96 figures for the financial cost of stress have been used in the model as these were the figures provided by the HSE in the document. More recent figures provided by other organizations suggest that these are an underestimate, but the more conservative financial costs provided by the HSE were chosen to avoid any discord.

The HSE recommended a 5-step stress risk assessment that focuses on assessing and then addressing 7 major hazards: Culture of the organization and how it deals with stress (for example, culture of long hours of work); demands: Exposure to physical hazards and workload (for example, volume and complexity of work; shift work); control: Employee involvement with how they do their work (for example, control balanced against demands); relationships include all work relationships (for example, bullying and harassment); change: Its management and communication to staff (for example, staff understanding why change is necessary); role: Employee understands role, and jobs are clearly defined (for example, conflicting roles avoided); support, training and factors pertaining to the individual: Support from peers and line managers, training for core functions of job, catering for individual differences.

The HSE assert that a proactive approach, as opposed to the more usual reactive approach, should be undertaken to tackle work-related stress. Therefore, the focus should be on stress prevention by assessing and subsequent removal of the hazards and not stress management, pressure management training or employee stress counseling. Qualitative assessment methods to find out whether work-related stress is a problem can include performance appraisals, informal discussions with staff, focus groups and return-to-work interviews. Quantitative methods include productivity data, sickness/absence data, staff turnover and questionnaires. However, the HSE do not recommend commercially available questionnaires as they may not be reliable or valid tests for work-related stress. The emphasis is on organizations developing their own audit tools with appropriate guidance. The risk assessment process in this document follows the principles explained in an earlier HSE publication.
